# 25-Hydroxyvitamin D, 1,25-Dihydroxyvitamin D, and Peripheral Bone Densitometry in Adults with Celiac Disease

**DOI:** 10.3390/nu12040929

**Published:** 2020-03-27

**Authors:** Carolina Ciacci, Giancarlo Bilancio, Ilaria Russo, Paola Iovino, Pierpaolo Cavallo, Antonella Santonicola, Cristina Bucci, Massimo Cirillo, Fabiana Zingone

**Affiliations:** 1 Celiac Center at Department of Medicine, Surgery, Dentistry, Scuola Medica Salernitana, University of Salerno, 84126 Salerno, Italy; russoilaria3@gmail.com (I.R.); piovino@unisa.it (P.I.); antonellasantonicola83@gmail.com (A.S.); cristinabucci@hotmail.it (C.B.); 2 Nephrology Unit, AOU San Giovanni di Dio e Ruggi d’Aragona, 84126 Salerno, Italy; giancarlo.bilancio@gmail.com; 3 Department of Physics, University of Salerno, 84126 Salerno, Italy; pcavallo@unisa.it; 4 ISC-CNR, Istituto Sistemi Complessi del CNR, 00185 Rome, Italy; 5 Department of Public Health, University of Naples Federico II, 80138 Naples, Italy; mcirillo@unisa.it; 6 Gastroenterology Unit, Department of Surgery, Oncology and Gastroenterology, University of Padua, 35128 Padua, Italy

**Keywords:** celiac disease, vitamin D, bone mineral density, peripheral quantitative computed tomography, parathyroid hormone

## Abstract

**Background**: Adults with celiac disease (CeD) show low bone mineral density (BMD) and high fracture risk. CeD guidelines suggest measurements of serum minerals and vitamin D. However, studies on vitamin levels in CeD patients are contradictory. **Aim**: To investigate in CeD, 25-hydroxy-vitamin D [25(OH)D], 1,25-dihydroxy-vitamin D [1,25(OH)2D], and related analytes and to evaluate their relationships to peripheral BMD as assessed by peripheral quantitative computed tomography (pQCT). **Methods**: Gluten-free diet (GFD)-treated, and untreated adult CeD patients naïve to vitamin D and calcium supplementation underwent measurements of serum 25(OH)D, 1,25(OH)2D, parathyroid hormone (PTH), total calcium, phosphate, and of radius BMD by pQCT. **Results**: Complete data were collected in 105 patients for lab tests and 87 patients for BMD. For lab tests, untreated CeD differed from treated CeD for 22.0% lower serum 25(OH)D (*p* = 0.023), 42.5% higher serum PTH (*p* < 0.001), and 13.0% higher serum 1,25(OH)2D (*p* = 0.029) in the presence of similar serum calcium and phosphorus (*p* > 0.35). For BMD, untreated CeD differed from treated CeD for lower diaphyseal cortical BMD (1133 and 1157 mg/cm^3^, *p* = 0.004) but not for distal BMD (total, trabecular, and subcortical, *p* > 0.13). Independent correlates of diaphyseal cortical BMD were GFD treatment and body mass index (*p* < 0.05). **Conclusions**: Data indicated that, compared to CeD patients on a gluten-free diet, untreated adult CeD patients at diagnosis had lower 25(OH)D, higher PTH, and higher 1,25(OH)_2_D in the absence of difference in serum calcium and phosphorus. 25(OH)D and 1,25(OH)_2_D, even below the normal range, were not associated with BMD. Our findings do not support the use of vitamin D supplementation for all CeD adults.

## 1. Introduction

Adult patients affected with celiac disease (CeD) have a low bone mineral density (BMD) and a higher risk of bone fracture compared with the general population [1,2]. With regard to these disorders, CeD guidelines suggest measuring calcium, alkaline phosphatase, and vitamin D levels at CeD diagnosis and the supplementation of vitamin D and/or calcium in the case of deficiency [3]. The prevalence and the importance of vitamin D and calcium deficiency in CeD have been debated [4]. Recent studies reported conflicting results about serum vitamin D levels in CeD patients at diagnosis and on a gluten-free diet (GFD) [5,6,7,8]. An essential source of confounding is the complexity of vitamin D metabolism that involves at least four different levels. First, the level of inactive, nonhydroxylated forms of vitamin D that can be generated from the skin after sun exposure and/or absorbed from a few vitamin D-rich foods [9]. Second, the hepatic hydroxylation of these nonhydroxylated forms that generates 25-hydroxy-vitamin D [25(OH)D], a molecule which has limited biological activity [10]. Third, the hydroxylation of 25(OH)D, which occurs mainly in the kidneys after stimulation of the parathyroid hormone (PTH), generates 1,25-dihydroxy-vitamin D [1,25(OH)_2_D], the molecule which has the highest biological activity. Fourth, the inactivation of 1,25(OH)_2_D via transformation in 24,25(OH)_2_-vitamin D or other catabolites [11]. Many studies have investigated 25(OH)D in CeD, but only a few reported data on other forms of vitamin D [12]. Also, previous studies have reported conflicting findings on the role of 25(OH)D levels on BMD in CeD [12]. This has mostly been evaluated by the dual-energy X-ray absorptiometry (DEXA), while authors have recently used peripheral quantitative computed tomography (pQCT) [13,14]. The assessment of peripheral BMD by pQCT has received little attention, although the technique implies shallow radiation exposure and gives separate and accurate measures of the trabecular, subcortical, and cortical bones [15]. The International Society for Clinical Densitometry stated that pQCT is in rapid development as an accurate method for fracture risk assessment, diagnosis, treatment initiation, and monitoring BMD for the clinical evaluation of osteoporosis [16]. Therefore, the present study aimed to investigate the serum levels of 25(OH)D and 1,25(OH)_2_D, minerals, and PTH in adult patients with untreated or treated CeD and to evaluate the correlation of these markers to peripheral BMD as assessed by pQCT.

## 2. Methods

The Ethical Committee of the University of Salerno approved the present study (protocol # 44 of 23-6-2015). We consecutively and prospectively enrolled all adults newly diagnosed with CeD (untreated) or on regular treatment with GFD (treated) who referred from October 2015 to March 2017 to our Celiac Unit of the University Hospital in Salerno. For untreated CeD, inclusion criteria were the absence of previous CeD diagnosis, positive serology for CeD (antitransglutaminase and antiendomysium), and the evidence of intestinal mucosal damage. For treated CeD, inclusion criteria were the report of a previous CeD diagnosis and proper compliance to treatment with GFD with negative CeD serology at follow-up. For both groups, exclusion criteria were the refusal or the withdrawal of the informed consent; age below 18 or over 65 years; past or current treatment or supplementation with any form of vitamin D, fish oil, and/or calcium salts; severe kidney or liver disease; mental illness; pregnancy and/or breastfeeding; use of drugs interfering with calcium homeostasis; or alcohol abuse. Data collection included the date of visit, anthropometry, symptoms of CeD at diagnosis classified according to the Oslo definition [17], habitual time of moderate to vigorous recreational physical activity per week [18], and results of lab tests. Moreover, the average local solar Global Horizontal Irradiance in the 30 days before blood withdrawal (from here on defined as solar irradiance [19] (MJ/m^2^ per day) to control for sunlight effects on vitamin D generation in the skin) was also evaluated. Lab tests were done in frozen serum stored at −80 °C and included 25(OH)D, 1,25(OH)_2_D, PTH, and total calcium, phosphate, and creatinine. All tests were done using fully automated assays. Architect assay (Abbott Diagnostics, Chicago, IL, USA) was used for the measurements of PTH, calcium, and phosphorus. The CKD-Epi equation was used for the calculation of estimated glomerular filtration rate (eGFR, mL/min x 1.73 m2), which was taken as an index of kidney function [20]. Serum 25(OH)D and 1,25(OH)_2_D were measured by chemiluminescent assay (Diasorin, Saluggia, Italy) [21]. As previously reported [13], and in accordance with a consensus report [22], the 25(OH)D chemiluminescent assay was recalibrated using the NIST-SRM 972a as the gold standard, that is, a product containing two levels of 25(OH)D certified by isotope dilution liquid mass chromatography (ID-LC-MS) and isotope dilution liquid chromatography-tandem mass spectrophotometry (ID-LC-MS/MS) [23]. Thus, the paper reported two sets of 25(OH)D data: Data obtained using the assay-specific calibration (defined as “nonrecalibrated data”) and data collected after recalibration with NIST-SRM 972a product (defined as “recalibrated data”). The deficiency of 25(OH)D was defined severe when serum 25(OH)D was < 10 ng/mL and mild-to-moderate when serum 25(OH)D was in the range 10–19 ng/mL [24]. The deficiency of 1,25(OH)_2_D was defined when serum 1,25(OH)_2_D was <18 pg/mL [25]. PTH was set high when ≥ 66 pg/mL [26].

Four different measurements of peripheral BMD as absolute mg/cm^3^ were performed using pQCT scans of the nondominant arm (StratecXCT 2000, StratecMedizintechnik GmbH, Pforzeim, Germany, software version 5.50d). Three measurements were taken at the distal radius for total BMD, trabecular BMD, and subcortical BMD (4% site). The fourth measurement was taken at the diaphyseal radius for assessment of cortical BMD (66% site). The Z-score of total BMD (that is, an index of BMD adjusted for sex and age) was calculated using device-specific population-based data of distal radius total BMD [27]. BMD was defined as normal when the Z-score was ≥ −1, with mild-moderate reduction when the Z-score was in the range from −1 to −2.49, and with severe reduction when the Z score was ≤ −2.50 [28].

### Statistical Analysis

Statistical procedures were performed using STATA version 11 and included ANOVA, chi-square analysis, McNemar test, Pearson correlation, and linear regression. Data were reported as prevalence (categorical variables) or as mean ± SD (continuous variables). A *p*-value < 0.05 was considered statistically significant.

## 3. Results

### 3.1. 25(OH)D, 1,25(OH)_2_D, PTH, and Other Lab Tests in CeD

We excluded one woman from Morocco, wearing a scarf and long-sleeved shirt in the summer, from the study. Table 1 reports descriptive statistics for the 105 patients with complete data for lab tests. All patients recruited were Caucasian, as expected, given the homogeneity of the population attending the hospital. The majority of patients were women treated with GFD (from now on defined as *treated*), without 25(OH)D deficiency, and without high serum PTH. Mild-to-moderate 25(OH)D deficiency was 1.25-times more prevalent using nonrecalibrated 25(OH)D data (McNemar test, *p* = 0.008), whereas the prevalence of severe 25(OH)D deficiency was identical in the two datasets. From here on, only recalibrated data are shown for 25(OH)D. Deficiency of 1,25(OH)_2_D was found in one untreated patient only. Mean serum PTH approached the threshold used for the definition of high serum PTH. 

Comparing untreated and treated patients, we observed no difference regarding sex (70% females in untreated and 80% in treated, *p* = 0.26), age (mean age 38.3 ± 11.8 untreated, 39.4 ± 11.5 treated, *p* = 0.6) and BMI (23.6 ± 3.5 untreated and 23.2 ±4.4 treated, *p* = 0.59). Table 2 summarizes the results of the comparison between untreated and treated patients. Untreated CeD differed from treated CeD for lower serum 25(OH)D, higher prevalence of 25(OH)D deficiency, higher serum PTH, higher prevalence of high PTH, and higher serum 1,25(OH)_2_D, but not from serum calcium and phosphorus. Findings were similar with adjustments for sex and age (Table S1). Also, findings were similar when analysis ANOVA of 25(OH)D was controlled for solar radiation (untreated CeD and treated CeD: 22.3 and 28.7 ng/mL, *p* = 0.023), when analysis of 1,25(OH)_2_D was controlled for eGFR (untreated CeD and treated CeD: 60.8 and 53.7 pg/mL, *p* = 0.028), and when analysis of serum total calcium was controlled for serum albumin (untreated CeD and treated CeD: 9.32 and 9.37 mg/dL, *p* = 0.615). 

**Figure 1:**
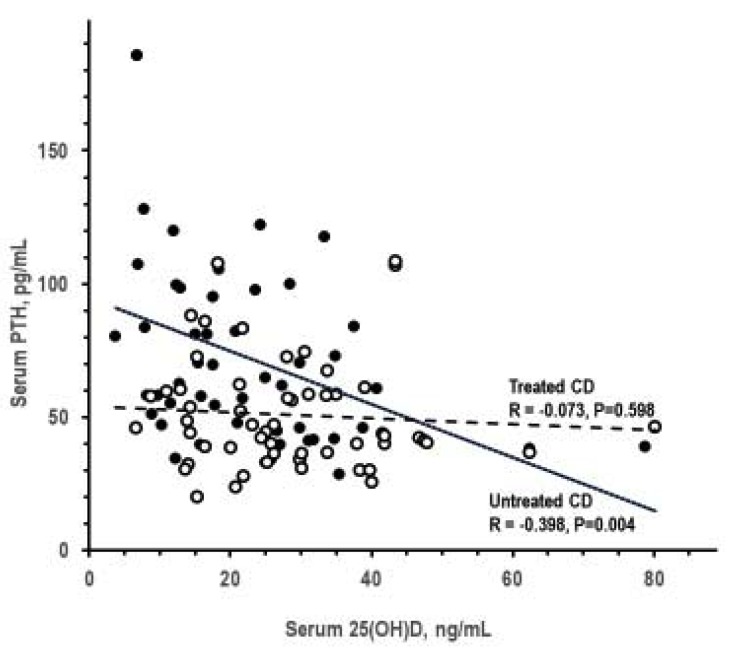
Pearson correlation between serum 25(OH)D and serum parathyroid hormone (PTH) in untreated CeD and treated CeD (closed and open symbols, respectively).

Table 3 summarizes the results of analyses by presence and degree of 25(OH)D deficiency. The 25(OH)D deficiency was associated with higher PTH, but not with alterations in other lab tests. Serum 25(OH)D inversely correlated with serum PTH in untreated CeD only (Figure 1). Analyses on the associations of 1,25(OH)_2_D deficiency could not be performed because only one patient had 1,25(OH)_2_D deficiency. Serum PTH and serum 1,25(OH)_2_D did not correlate significantly with each other in untreated CeD and treated CeD (*p* > 0.066).

### 3.2. Peripheral BMD in CeD

Complete pQCT data were collected in 87 patients because 18 patients refused to undergo pQCT. Table 4 reports descriptive statistics for the patients with complete pQCT data for radius BMD. The vast majority of patients were women treated with GFD and with some reduction of total BMD. 

Table 5 summarizes the results of analyses for comparisons of BMD between untreated and treated CeD. In univariate analysis, the difference between untreated CeD and treated CeD was not significant for distal radius total, trabecular, and subcortical BMD expressed in absolute units (mg/cm^3^). The difference was borderline significant for distal radius total BMD shown as Z-score (index controlled for sex and age) and significant for diaphyseal radius cortical BMD. The difference between untreated CeD and treated CeD was significant for distal radius total BMD also as absolute units when ANOVA was controlled for sex and age (untreated CeD and treated CeD: 298 and 324 mg/cm^2^, *p* = 0.035; complete data in Table S2).

Table 6 summarizes the results of analyses by tertile of diaphyseal radius cortical BMD. Tertile analysis was reported only for radius diaphyseal cortical BMD, since it was only the BMD parameter which was significant between untreated CeD and treated CeD (data in Table 5). 

In univariate analyses, cortical BMD was significantly associated with GFD treatment, sex, age, and serum PTH (Table 6). In multivariable regression of BMD over other variables (Table 7), independent correlates of diaphyseal radius cortical BMD were GFD treatment and body mass index (higher BMD in treated CeD and lower body mass index, respectively). For distal radius BMD, independent associations were found with subcortical BMD only for age (higher BMD in youngsters) and with total and trabecular BMD for sex and age (higher BMD in men and youngsters). Moreover, in multivariable linear regression analysis, older age was an independent risk factor of low total, trabecular, and subcortical BMD. Female gender was an independent risk factor for low total and trabecular BMD at the radius (Table 7). The analysis by tertile of cortical BMD was designed as the first approach in the investigation about correlates of cortical BMD reduction. Given that the literature did not report a reference range for cortical BMD, the definition of cortical BMD strata was based on data distribution within our study cohort. Compared to the multivariable analysis in Table 7, the tertile analysis of Table 6 gives more readable data about the characteristics of patients who had cortical BMD reduction. The lack of reference data for cortical BMD makes it impossible to add an appropriate reference. Finally, 25(OH)-vitD deficiency was not associated with the presence/absence of gastrointestinal symptoms at diagnosis or with the level of physical activity (data not shown). 

## 4. Discussion

Our study found that the prevalence of vitamin D deficiency in CeD patients was about 50% for 25(OH)D and about 1% for 1,25(OH)_2_D of CeD patients. The 25(OH)D deficiency was associated with a graded increase in serum PTH but not with alterations in serum 1,25(OH)_2_-D, calcium, or phosphorus. Compared with CeD patients on a GFD, adult untreated CeD patients at diagnosis had lower serum 25(OH)D, higher prevalence of mild-to-moderate 25(OH)D deficiency, higher serum PTH, higher prevalence of high PTH, and higher serum 1,25(OH)_2_D in the presence of similar levels of serum calcium and phosphorus groups. No data indicated an association of 25(OH)D deficiency or 1,25(OH)_2_D deficiency with low BMD. Untreated CeD and treated CeD differed for cortical BMD only. Last, multivariate analyses indicated that, besides GFD, lower BMI was also associated with higher cortical BMD in CeD patients. 

The results of the study supported the idea that the biologic activity of 25(OH)D is low and likely limited to an inhibitory effect of PTH secretion. The 25(OH)D deficiency was associated only with a graded increase in serum PTH. This association appeared without significant implications for BMD in the present series of CeD patients [12,29]. In turn, the higher serum PTH was a reasonable determinant of the higher serum 1,25(OH)_2_D given that the hydroxylation of 25(OH)D is stimulated by PTH [30]. The finding of sufficient 1,25(OH)_2_D, and of actually higher levels in untreated CeD, was intriguing because the strong intestinal effects of 1,25(OH)_2_D on calcium absorption could not be uniformly distributed along the intestine of CeD patients. Whereas mucosal damage in untreated CeD decreases in the craniocaudal direction and spares the distal tract of the intestine/ileum [31], calcium absorption and 1,25(OH)_2_D stimulation of calcium absorption normally occur at any intestinal segment throughout the ileum. Thus, calcium balance could be preserved in untreated CeD by higher levels of 1,25(OH)_2_D stimulating calcium absorption in the distal ileum, that is, in intestine segments, which are usually undamaged or less damaged in CeD. The observation of higher serum levels of PTH and 1,25(OH)_2_D in untreated CeD also implied the possibility that the stimulatory effects of PTH and 1,25(OH)_2_D on kidney tubule Ca reabsorption were the true determinants of the relative hypocalciuria classically reported in CeD [32,33]. Our findings, in contrast with the majority of the previous literature [34], did not support the idea that low BMD in CeD could depend on vitamin D deficiency and/or low intestinal calcium absorption. Other reasonable candidates could be malnutrition and/or inflammatory mechanisms [35] in accordance with a recent study [36]. In this view, the assessment of the active form of the vitamin D levels in CeD could change the perspective about the existence and the role of vitamin D deficiency in CeD as in other diseases. 

The main limitations of the present study were the number of patients and the fact that GFD effects were derived from the comparison between different groups. Regarding the second limitation, the present results were in good agreement with a pre- post-study in which BMD at DXA increased with the sole GFD [37]. The use of pQCT instead of DEXA could be considered another limitation. Nonetheless, compared with the traditional DEXA, pQCT has the advantage of providing accurate BMD measurements for total, cortical, subcortical, and trabecular bones [38]. Moreover, the use of DXA can imply a bias in shorter and/or lean individuals, who are often erroneously diagnosed as osteopenic or osteoporotic because of their small bones, a cofounder which could be important in CeD for the frequency of low anthropometry in CeD patients [39]. Last, previous studies demonstrated the utility of pQCT BMD measurements in premenopausal women with CeD and young patients with insulin-dependent diabetes mellitus and CeD [40]. In our study, we were not able to collect data regarding the different skin tones of our patients and the amount of time they usually spend outside for leisure. 

The merits of our study were the accurate selection of CeD patients, the fact that our series involved Caucasian people which belonged to a unique social group with a common national or cultural tradition, the accurate exclusion of patients with current or past supplementation of vitamin D, fish oil, and/or calcium [14,41], the use of the best available reference for calibration of the 25(OH)D assay [23], and the inclusion in the analysis of an accurate index of kidney function that could be relevant to 1,25(OH)_2_D generation and data interpretation [20].

Given that this is the first study using recalibrated assay for 25(OH)D in CeD, data for recalibrated 25(OH)D could not be compared to previous CeD data but only to general population data, in which a similar overestimate of mild-to-moderate 25(OH)D deficiency was reported for nonrecalibrated assay [13]. The lack of association between 25(OH)D deficiency and low BMD was in accordance with data reported in the general population [42,43]. The finding that 25(OH)D deficiency was not associated with low serum calcium or low serum phosphorous but only with high serum PTH ws also in accordance with the data in the general population [13]. 

The present finding was in good agreement with the report of the same group that found an increased BMD at DXA after GFD without any vitamin D supplementation [37]. 

In conclusion, data indicated that, compared to CeD patients on GFD, untreated CeD adults at diagnosis had lower 25(OH)D, higher PTH, and higher 1,25(OH)_2_ in the presence of similar serum calcium and phosphorus. Untreated CeD patients also had lower cortical BMD at the peripheral level as assessed by pQCT. Patients’ vitamin D status was not associated with BMD. Therefore, the findings do not support the use of vitamin D supplementation to all adult CeD. The results are in accordance with the guidelines of the American College of Physicians for BMD and osteoporosis about the uncertain efficacy of vitamin D or calcium supplementation [44]. 

## Figures and Tables

**Table 1:** Descriptive statistics for patients with lab tests: Mean ± SD or prevalence.

Number of Patients	105
Women, %	75.2%
Age, years	39.0 ± 11.3
on GFD, %	52.4%
Weight, kg	64.6 ± 14.0
Height, cm	166 ± 9
Body mass index, kg/m^2^	23.4 ± 4.0
Sun radiation in the month of blood withdrawal, MJ/m^2^	14.1 ± 5.8
Serum Vitamin D	
Non-recalibrated 25(OH)D, ng/mL	22.0 ± 9.6
with mild-moderate deficiency (10–19 ng/mL), %	34.3%
with severe deficiency (<10 ng/mL), %	10.5%
Recalibrated 25(OH)D, ng/mL	25.6 ± 14.3
with mild-moderate deficiency (10–19 ng/mL),%	27.6%
with severe deficiency (<10 ng/mL), %	10.5%
1,25(OH)_2_D, pg/mL	57.1 ± 16.5
with deficiency (<18 pg/mL), %	1.0%
Serum PTH, pg/mL	61.1 ± 30.6
with high PTH (≥ 66 pg/mL)	31.4%
Serum phosphorus, mg/100 mL	3.45 ± 0.60
Serum total calcium, mg/100 mL	9.35 ± 0.72
Serum albumin, g/100 mL	4.47 ± 0.43
Albumin-normalized serum calcium, mg/100 mL	9.44 ± 0.70
Serum creatinine, mg/100 mL	0.70 ± 0.15
eGFR, mL/min × 1.73 m²	110 ± 15

**Table 2:** Lab test comparisons between untreated and treated celiac disease (CeD): Mean or prevalence.

	Untreated CeD	Treated CeD	*p* *
Number of Patients	50	55	
Recalibrated 25(OH)D, ng/mL	22.3	28.6	0.023
with mild-moderate deficiency, %	32.0%	23.6%	0.018
with severe deficiency, %	18.0%	3.6%	
Serum PTH, pg/ mL	72.4	50.8	<0.001
with high PTH, %	46%	18.2%	0.002
1,25(OH)_2_D, pg/mL	60.8	53.8	0.029
with deficiency, %	2.0%	0.0%	0.292
Serum total calcium, mg/dL	9.33	9.36	0.797
Serum phosphorus, mg/dL	3.51	3.40	0.356

* by ANOVA or chi-square.

**Table 3:** 25(OH)D status and other lab tests: Mean.

	25(OH)D Status			*p* for Trend *
	Normal	Mild Deficiency	Severe Deficiency	
Serum PTH, pg/mL	53.2	65.9	94.6	<0.001
1,25(OH)_2_D, pg/mL	56.7	58.2	56.6	0.860
Serum total calcium, mg/dL	9.38	9.26	9.39	0.770
Serum phosphorus, mg/dL	3.44	3.45	3.51	0.759

* by ANOVA or chi-square.

**Table 4:** Descriptive statistics for the 87 patients with peripheral quantitative computed tomography (pQCT) assessment of radius bone mineral density: Mean ± SD or prevalence.

Number of Patients	87
Women, %	75.9%
Age, years	39.4 ± 11.1
on GFD, %	52.9%
Weight, kg	65.8 ± 14.2
Height, cm	166 ± 9
Body mass index, kg/m^2^	23.8 ± 4.1
Distal radius total BMD, mg/cm^3^	312 ± 60
Z-score	−1.38 ± 0.95
with mild-moderate BMD reduction,%	52.9%
with severe BMD reduction, %	14.9%
Distal radius trabecular BMD, mg/cm^3^	182 ± 43
Distal radius subcortical BMD, mg/cm^3^	422 ± 86
Diaphyseal radius cortical BMD, mg/cm^3^	1145 ± 40

**Table 5:** Differences in bone mineral density (BMD) between untreated CeD and treated CeD: Mean or prevalence.

	Untreated CeD	Treated CeD	*p* *
Number of Patients	41	46	
Distal radius total mineral density, mg/cm^3^	301	321	0.137
Z-score	−1.59	−1.20	0.055
with mild-moderate BMD reduction, %	56.1%	50.0%	
with severe BMD reduction, %	19.5%	10.9%	0.259
Distal radius trabecular mineral density, mg/cm^3^	179	185	0.539
Distal radius subcortical mineral density, mg/cm^3^	410	431	0.258
Diaphyseal radius cortical BMD, mg/cm^3^	1133	1157	0.004

* by ANOVA or chi-square.

**Table 6:** Analysis by tertile of diaphyseal radius cortical BMD: Prevalence or mean of gluten-free diet (GFD), sex, age, body mass index, and serum concentrations of 25(OH)D, PTH, 1,25(OH)_2_D, calcium, and phosphorus.

	Diaphyseal Radius Cortical BMD, mg/cm^3^			*p* for Trend
	<1135	1135–1166	>1166	
Number of Patients	29	30	28	
On GFD, %	31.0%	63.35	64.3%	0.012
Women, %	65.5%	73.3%	89.3%	0.038
Age, y	43.2	37.8	37.0	0.034
Body mass index, kg/m^2^	25.5	23.4	22.5	0.005
Recalibrated 25(OH)D, ng/mL	24.4	28.1	25.7	0.738
with mild-moderate deficiency, %	34.5%	23.3%	21.4%	
with severe deficiency, %	13.8%	3.3%	17.9%	0.775
Serum PTH, pg/mL with high PTH, %	73.1 44.8%	55.4 26.7%	46.7 10.7%	0.001 0.004
1,25(OH)_2_D, pg/mL with deficiency, %	59.8 0.0%	58.8 0.0%	50.8 3.6%	0.051 0.211
Serum total calcium, mg/100 mL	9.45	9.43	9.28	0.409
Serum phosphorus, mg/100 mL	3.53	3.44	3.39	0.416

* by ANOVA or chi-square.

**Table 7:** Multivariable linear regression of radius BMD (dependent variable) regressed over GFD treatment, sex, age, body mass index, and lab variables: Standardized regression coefficient.

Independent Variables	Dependent Variable			
	Distal Radius Total BMD	Distal Radius Trabecular BMD	Distal Radius Subcortical BMD	Diaphyseal Radius Cortical BMD
Treatment with GFD, yes/no = 1/0	0.126 ^ns^	0.027 ^ns^	0.097 ^ns^	0.246 *
Sex, M/W = 1/0	0.250 *	0.322 **	0.140 ^ns^	−0.009 ^ns^
Age, years	−0.292 **	−0.335 **	−0.234 *	−0.163 ^ns^
Body mass index, kg/m^2^	0.051 ^ns^	0.088 ^ns^	0.062 ^ns^	−0.278 **
Serum 25(OH)D, ng/mL	0.065 ^ns^	0.125 ^ns^	0.031 ^ns^	−0.169 ^ns^
Serum 1,25(OH)_2_D, pg/mL	−0.048 ^ns^	−0.088 ^ns^	−0.011 ^ns^	−0.044 ^ns^
Serum PTH, pg/mL	−0.155 ^ns^	−0.108 ^ns^	−0.143 ^ns^	−0.189 ^ns^
Serum total calcium, mg/100 mL	0.045 ^ns^	−0.186 ^ns^	−0.003 ^ns^	0.050 ^ns^
Serum phosphate, mg/100 mL	−0.110 ^ns^	−0.198 ^ns^	−0.102 ^ns^	−0.098 ^ns^

^ns^ not significant, *p* > 0.05. * *p* ≤ 0.05, ** *p* ≤ 0.01.

## Supplementary Materials

The following are available online at https://www.mdpi.com/2072-6643/12/4/929/s1, **Table S1** Lab test comparison between untreated CeD and treated CeD in ANOVA adjusted for sex and age: Means; **Table S2**—Radius BMD in untreated CeD and treated CeD in ANOVA adjusted for sex and age: Mean.
