# Supplementary material for: 25-Hydroxyvitamin D, 1,25-Dihydroxyvitamin D, and Peripheral Bone Densitometry in Adults with Celiac Disease
Source: Nutrients. 2020 Mar 27;12(4):929. doi: 10.3390/nu12040929 (PMC7231255; doi:10.3390/nu12040929)
Supplement: Supplementary file 1 [file nutrients-12-00929-s001.pdf]

Table S1 - Lab test comparison between untreated CeD and treated CeD in ANOVA adjusted for sex and age: means.

	Untreated CeD	Treated CeD	
Number of patients	50	55	*P**
Recalibrated 25-hydroxy-vitamin D, ng/mL	22.2	28.7	*0.019*
Serum PTH, pg/ mL	72.2	51.0	*<0.001*
1,25(OH_2)-vitamin D, pg/mL	60.9	53.6	*0.025*
Serum total calcium, mg/dL	9.33	9.37	*0.778*
Serum phosphorus, mg/dL	3.52	3.39	*0.239*

* by ANOVA

Table S2 – Radius BMD in untreated CeD and treated CeD in ANOVA adjusted for sex and age: mean.

	Untreated CeD	Treated CeD	
Number of patients	41	46	*P**
Distal radius total mineral density, mg/cm^3	298	324	*0.035*
Distal radius trabecular mineral density, mg/cm^3	176	188	*0.155*
Distal radius subcortical mineral density, mg/cm^3	407	434	*0.137*
Diaphyseal radius cortical BMD, mg/cm^3	1132	1158	*0.002*

* by ANOVA
